# Reduced Body Mass in a Highly Insectivorous Mammal, the Garden Dormouse—Ecological Consequences of Insect Decline?

**DOI:** 10.1002/ece3.71340

**Published:** 2025-04-21

**Authors:** Stefanie Erhardt, Marc I. Förschler, Joanna Fietz

**Affiliations:** ^1^ Department of Zoology, Institute of Biology University of Hohenheim Stuttgart Germany; ^2^ KomBioTa – Center of Biodiversity and Integrative Taxonomy University of Hohenheim Stuttgart Germany; ^3^ Department for Ecological Monitoring, Research and Species Protection Black Forest National Park Seebach Germany

**Keywords:** body condition, conservation, physiological indicators, rodent, stress

## Abstract

Biodiversity is decreasing worldwide, and early indicators are needed to identify endangered populations before they start to decline in abundance. In mammals, body mass (BM) is regarded as an indicator of fitness, and its loss is used as an early warning signal preceding population decline. The garden dormouse ( *Eliomys quercinus* , Gliridae, BM: 60–110 g) is a small mammalian hibernator that has disappeared from over 50% of its former range in the last decades. The aim of this study was to investigate whether garden dormice from a presumably thriving and stable population already show early warning signals, which may precede a population decline. We therefore conducted capture‐mark‐recapture studies during 2003–2005 (Period 1) and 2018–2021 (Period 2) in the Northern Black Forest, one of its last natural distribution areas in Germany. We collected fecal samples, measured BM, and tibia length as a proxy for size and age. Results revealed that in Period 2 adult dormice had a significantly lower (12%) pre‐hibernation BM, corrected for body size, and juveniles showed a significantly lower BM gain after weaning than nearly two decades ago. Fecal samples collected in Period 2 showed that arthropods represented the main food residues in fecal samples during juvenile growth and pre‐hibernation fattening. Ambient temperature during hibernation showed no correlation with BM at emergence. We could not detect a phenological time shift in reproduction; however, we found only one birth peak in Period 2, compared with two birth peaks in Period 1. Observed changes in BM and reproduction pattern represent early warning signals, as they point to an insufficient availability of high‐quality food, which prevents dormice from meeting their nutritional requirements, with potentially serious consequences for their reproductive success and survival. As arthropods are the dominant food resource, their decline may at least partly explain this phenomenon.

## Graphical abstract

In this study, we investigated the threatened garden dormouse ( *Eliomys quercinus* ) in one of its last natural distribution areas in Germany. We found a decrease in pre‐hibernation body mass, reduced juvenile growth, and a change in reproductive pattern compared with 20 years ago, which is likely to affect their survival and reproductive success. These changes are probably caused by the decline of arthropods, which were found to be the main food resource during these critical life history stages.

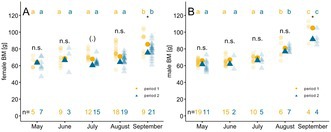

## 1 Introduction

Biodiversity is declining worldwide (IPBES 2019) with almost 30% of the world's species threatened with extinction (IUCN 2022). This decline is caused by human‐induced pressures through intensified land use and subsequent habitat destruction and fragmentation, climate change, exploitation, pollution, and invasive species (Hochkirch et al. 2023; Jaureguiberry et al. 2022). On a population level, the effects of these deleterious impacts can only be detected when populations start to decline. Therefore, there is a need for indicators that help ecologists to detect if a population is at risk before it shows a decline in abundance (Ellis et al. 2012). One approach is to measure traits that indicate whether individuals are facing environmentally challenging periods and that are good proxies for fitness (Bergman et al. 2019; Cerini et al. 2023; Ellis et al. 2012). In mammals, such indicators can include prolonged elevated levels of stress hormones (e.g., glucocorticoids, Havenstein et al. 2021) and changes in behavior (e.g., shift in diet, timing of reproduction) or morphometric traits (e.g., body mass loss; Cerini et al. 2023; Clements et al. 2017; Ellis et al. 2012).

Sufficient energy reserves stored as body fat help individuals counter stress‐inducing life events and buffer the impacts of environmental stressors (Bright Ross et al. 2021). In ecological studies, body mass (BM) is therefore often used as a proxy for the availability of energy stores and is thought to be associated with survival and reproductive success (e.g., Bright Ross et al. 2021; Plard et al. 2015; Rughetti and Ferloni 2023). However, BM alone is strongly influenced by an individual's body size (Balčiauskas et al. 2015). Therefore, ecologists often use body condition indices to account for variations in body size and to better assess an individual's energy reserves (i.e., fat or lean mass; Peig and Green 2010; Schulte‐Hostedde et al. 2005). Depending on what kind of data can be measured in the field, different body condition indices are used (Labocha et al. 2014). However, studies show that these body condition indices do not necessarily correlate well with available energy reserves and, in some cases, are no better predictors of energy reserves than BM alone (McGuire et al. 2018; Wishart et al. 2024). Thus, depending on the life history of the species and which body measurements can be taken, researchers must decide which proxy is the most informative for their investigations. Reproduction represents an energy‐consuming and challenging life history stage, in particular for female mammals (Clutton‐Brock et al. 1989; Speakman 2008). Reproductive success has been shown to be related to BM, with females in better body condition being more likely to become pregnant, e.g., North American elk ( *Cervus canadensis* ; Morano et al. 2013), have more offspring, e.g., yellow‐necked mice ( *Apodemus flavicollis* ), tundra voles (*Alexandromys oeconomus*; Balčiauskas et al. 2022) and alpine marmots ( *Marmota marmota* ; Tafani et al. 2013), produce offspring with a more powerful immune system and higher survival rates, e.g., roe deer ( *Capreolus capreolus* ; Cheynel et al. 2021; Plard et al. 2015; but see Keane et al. 2007), or have an increased reproductive success, e.g., northern elephant seals ( *Mirounga angustirostris* ; Beltran et al. 2023). But also in mammalian males, BM positively influences reproductive success, with heavier‐born males having a higher lifetime reproductive success than lighter ones (Kruuk et al. 1999). In male alpine marmots, reproductive activity was positively correlated with the preceding pre‐hibernation BM (Arnold and Dittami 1997).

In seasonal environments, animals can periodically experience stressful events such as food scarcity or low ambient temperature (Bright Ross et al. 2021; Humphries et al. 2002; Schorr et al. 2009). In mammals, these challenges have been shown to result in a decreased body condition (body fat score), which is associated with higher susceptibility to pathogens and a reduced survival probability (Beldomenico et al. 2009; Beldomenico and Begon 2010). In bighorn sheep ( *Ovis canadensis* ), infected individuals had lower body fat levels, affecting survival, reproduction, and overall fitness (Smiley et al. 2024). In European badgers ( *Meles meles* ), unfavorable weather conditions and low food availability led to increased energy expenditure, low body condition, and reduced survival probability (Bright Ross et al. 2021). Also, environmental changes such as habitat destruction and fragmentation can lead to a decline in BM because of reduced availability of food resources and more energy‐consuming foraging periods (Pagano et al. 2018; Seltmann et al. 2017). Energy reserves stored as body fat can be assumed to mitigate these effects and increase survival during these seasonal or environmental challenges. Thus, in mammals, BM or BM indices represent good indicators of reproductive success, survival, and therefore fitness. The occurrence of a reduced BM can be regarded as an early indicator of a population being at risk before it shows a decline in abundance.

The garden dormouse ( *Eliomys quercinus* ) is a small (60–110 g) hibernating mammal of the family Gliridae (Vaterlaus 1998). Originally, it occurred from Portugal to the Urals. However, in the last 20–30 years, it has disappeared from more than 50% of its former range, mainly in Central and Eastern Europe. It is regarded as extinct mainly in the eastern parts of its former range (Bertolino et al. 2024). The nocturnal garden dormouse is considered to be the fastest declining mammal in Europe. On the IUCN (International Union for Conservation of Nature) Red List of Threatened Species, it is listed as vulnerable (Bertolino et al. 2024). Reasons for this decline may include habitat destruction, limited access to essential resources, and genetic isolation due to habitat fragmentation (Meinig and Büchner 2012). However, in addition to other environmental impacts, climate change could also potentially be regarded as a serious threat to this species (Fietz et al. 2020; Giroud et al. 2023; Wells et al. 2022). In Central Europe, the garden dormouse enters hibernation during winter (October until April), when energy demands increase due to high thermoregulation costs while food availability is limited (Ruf and Geiser 2015). As a fat‐storing hibernator, the garden dormouse accumulates body fat shortly before the onset of hibernation (Vaterlaus 1998). Winter survival depends on body fat accumulated before the onset of hibernation, the duration of hibernation, and the energy consumption during hibernation (Humphries et al. 2002; Murie and Boag 1984). Hibernators have a species‐specific optimal body temperature (*T*_b_) during hibernation, at which energy expenditure is at a minimum. Ambient temperature (*T*_a_) below or above this optimum leads to an increase in energy consumption (Buck and Barnes 2000; Geiser 2004). During warm winters, i.e., due to climate change, the energy consumption of hibernators is elevated, and they lose BM faster, which was shown to lead to an earlier emergence and/or a lower BM at emergence, which may affect their survival and timing of reproduction (Fietz et al. 2020; Findlay‐Robinson et al. 2023; Turbill and Prior 2016; reviewed Wells et al. 2022). The time immediately after emergence is a critical period in the yearly cycle of hibernators, as food availability is still limited, and hibernators have to restore their regressed organs (Bieber et al. 2011; Carey 1990; Hume et al. 2002; Inouye et al. 2000). After emergence, edible dormice ( *Glis glis* ) show a further reduction in their BM, immune defense is impaired, and seasonal survival rates are lowest (Havenstein et al. 2016; Lebl et al. 2011).

The garden dormouse is omnivorous and feeds on a variety of food resources, such as seeds, fruits, nuts, and buds, but has a preference for a diet of animal origin, such as arthropods, in particular insects (Gil‐Delgado et al. 2010; Kuipers et al. 2012; Dooren et al. 2024). Generally, insects represent a high‐quality food as they are rich in protein and fat and provide a balanced diet (Costello et al. 2016; Rothman et al. 2014). During recent decades, a drastic decline in insect biomass (more than 75%) and diversity has been observed (e.g., Hallmann et al. 2017; Sánchez‐Bayo and Wyckhuys 2019; Staab et al. 2023). This decline can have profound consequences for food webs, ecosystem functions, and therefore, whole ecosystems (Duffy et al. 2007; Hooper et al. 2005). For species that rely on insects as the main food resource, drastic insect decline can have serious consequences for their survival and reproductive success, especially when insect food is needed in critical life history stages (Grames et al. 2023; Møller 2019).

The aim of this study was to investigate whether garden dormice of a presumably thriving and stable population in western Germany already show early warning signals that may precede a population decline. These signals could be associated with severely reduced availability of food resources due to insect decline and with climate change.

Specifically, we asked the following questions:

1. Do garden dormice show a decline in BM, corrected for body size (as a proxy for fitness), during critical life history stages such as pre‐hibernation fattening, reproduction, and juvenile growth?
2. Do insects represent an important food resource during these critical life history stages?
3. Do garden dormice have a reduced BM, corrected for body size, after emerging from hibernation, which can be explained by winter *T*_a_?
4. Do garden dormice show a change in their reproductive pattern?

To answer these questions, we analyzed data from capture‐mark‐recapture studies conducted in the Northern Black Forest during 2003–2005 and 2018–2021. The Black Forest is one of the last distribution areas of garden dormice within mountainous, coniferous‐rich forests in Germany (Büchner et al. 2024). We measured the tibia length as a proxy for body size in adults and for age in juveniles, as well as BM of each capture. We collected fecal samples in 2018 and 2019 and semi‐quantitatively analyzed food residues.

## 2 Materials and Methods

### 2.1 Study Animal

Garden dormice have a head‐to‐body length of up to 17 cm, and BM can reach 130 g shortly before hibernation (Vaterlaus 1998). The final body size is reached after 2 years (Stumpfel et al. 2017). In the wild, garden dormice can reach an age of up to 3–4 years (Bertolino et al. 2001). In Central Europe, the main natural habitats are coniferous mixed forests (Bertolino 2017). The occurrence and length of the hibernation period depend on the geographical location. While hibernation in Central Europe lasts from October to April, garden dormice in the Mediterranean area may remain active throughout the winter (Bertolino et al. 2001; Moreno 1988; Vaterlaus 1998). Reproduction starts directly after hibernation. Females give birth after 23 days, with litters averaging 4–6 young, which are weaned after 34–36 days (Moreno and Collado 1989; Schlund 2005; Vaterlaus 1998).

### 2.2 Study Site

Our three study sites were located in the coniferous mixed forest of the Northern Black Forest, Southwest Germany. Ruhesteinloch (RL) and Waldklassenzimmer (WK) were located within the Black Forest National Park in close proximity to each other (1 km). In both study sites, the dominant tree species are spruce ( *Picea abies* ) with silver fir ( *Abies alba* ) and a few beech trees ( *Fagus sylvatica* ). The study site Melkerei (ME) was located outside the western border of the National Park and is dominated by beech and spruce. The understory in all sites was dominated by blueberry ( *Vaccinium myrtillus* ). The study sites contained between 60 and 72 nest boxes (Schwegler 3SV) mounted on trees 1.5 m above the ground, 25 m apart, at the nodes of a rectangular grid (Table 1).

**TABLE 1:** Coordinates, elevations, and sizes of the three study sites in the Northern Black Forest.

Study site	Coordinates	Elevation [m a.s.l]	Size [ha]
RL	48.566° N, 8.222° E	820–860	4.3
WK	48.561° N, 8.230° E	900–930	4.7
ME	48.546° N, 8.200° E	780–820	3.4

### 2.3 Capture‐Mark‐Recapture

RL and ME were monitored during 2003–2005 (Period 1) and 2018–2021 (Period 2, Table 2); however, no garden dormice were captured during monthly nest box monitoring in ME during Period 2. Therefore, we newly established the study site WK in 2019, where garden dormice were known to occur. WK was monitored from 2019 until 2021. Monitoring was carried out between May and October at weekly (2004, 2005) and monthly (2003, 2018–2021) intervals. First captured garden dormice were marked individually (ID) using subcutaneously implanted passive integrated transponders (Trovan, EURO I.D. Usling GmbH, Germany) or by individual ear markings (Table 2). BM was measured to the nearest g by using a 100 g or 300 g spring balance (Pesola, divisions: 1 g and 2 g, respectively, precision: 99.7%). Tibia length (TL) was measured with a sliding caliper to the nearest 0.1 mm as a proxy for body size. In juveniles, TL was used to determine age, taking weekly TL measures of a litter of 5 juveniles born on July 8, 2005 as a reference. Of each captured garden dormouse, sex and age class (juvenile: born within the same year; adult: after first hibernation) were determined. Capture and treatments were carried out under the license of the Regierungspräsidium Freiburg (56–8852.44; G‐18–22) and the Black Forest National Park.

**TABLE 2:** Number of garden dormice captures (*n*) and number of captured individuals (*N*, bold) of adult females, adult males, juveniles, and total number for the three different study sites, time periods, and years of capture.

Study site	Time period	Years of capture	Fmale		Male		Juvenile		Total	
			*n*	*N*	*n*	*N*	*n*	*N*	*n*	*N*
RL	1	2003–2005	37	**10**	52	**13**	53	**44**	142	**67**
	2	2018–2021	50	**12**	25	**16**	77	**56**	152	**84**
ME	1	2003–2005	16	**3**	2	**1**	17	**9**	35	**13**
	2	2018–2019	0	**0**	0	**0**	0	**0**	0	**0**
WK										
	2	2019–2021	15	**4**	4	**2**	14	**12**	33	**18**
		Total	118	**29**	83	**32**	161	**121**	362	**182**

*Note:* 0 indicates that nest boxes were monitored, but no garden dormice were found.

### 2.4 Dietary Analysis

During Period 2, fecal samples were collected from each individual that defecated during handling. The samples were transferred to 1.5 mL tubes with 70% EtOH and stored at 4°C. For analysis, each sample was dissolved with 70% EtOH in a Petri dish placed on millimeter paper and evenly distributed and inspected using a stereoscopic microscope (10 × 0.65–5). The food residues were identified using the morphological characteristics of reference plant samples collected at the study sites and classified into different categories: arthropods, fruits, seeds, blossoms, buds, leaves, and moss (Table 3). The proportion of each category within the sample was quantified by determining the area within the Petri dish in mm^2^ and calculated as % of the total. Food residues that could not be assigned to a category were classified as miscellaneous. To quantify the importance of different food residues for the different months, we also calculated the frequency of occurrence of the main food residues (arthropods, fruits, and seeds) in juvenile and adult fecal samples collected during the respective months (Dunlop et al. 2017).

**TABLE 3:** Food categories, different food items, and structure identified in the food residues within the fecal samples of garden dormice.

Food category	Food items consumed	Structure identified
Arthropods	Arthropods	Chitin parts, whole arthropods
Fruits	Blackberry (*Rubus* spp.) Wild strawberry ( *Fragaria vesca* ) Rowan ( *Sorbus aucuparia* ) Blueberry ( *Vaccinium myrtillus* )	Fruit seeds, exocarp Fruit seeds Fruit seeds Fruit seeds, exocarp
Seeds	Beech fruits ( *Fagus sylvatica* ) Grass fruits Coniferous cones	Husk, seed coat Husk, seed coat Strobilus
Blossoms	Blossoms	Pistil
Buds	Buds	Bud scales
Leaves	Leaf buds	Leaf parts, leaf vein
Moss	Moss	Moss gametophyt

### 2.5 Ambient Temperature

We used daily minimum *T*_a_ recordings, measured 2 m above ground, from the weather station Freudenstadt of the Deutscher Wetterdienst (DWD; dwd.de), located 18 km southeast of the study sites at an altitude of 797 m a.s.l. We took the median of all daily minimum *T*_a_ recorded during the hibernation period (October to April) as a measure for the minimum *T*_a_ during hibernation (*T*_aminhib_).

### 2.6 Statistical Analyses

To investigate whether the proportions of seeds and arthropods differed among the active season in adults, we conducted linear mixed‐effect models for seeds and arthropods separately with the “lmer” function from package lme4, extended by package lmertest with Satterthwaite approximation for degrees of freedom (Bates et al. 2015; Kuznetsova et al. 2017). For the analysis, we included year (2 levels: 2018, 2019), sex, and month (4 levels: May/June, July, August, September) as covariates. ID was included as a random factor. Because of small sample sizes, fecal samples collected in May (*n* = 2) and June (*n* = 1) were pooled. Note that fruits could only be detected in fecal samples of adults in August. To analyze the variation of arthropod residues in fecal samples of weaned juveniles, we included year (2 levels: 2018, 2019) and month (3 levels: July, August, September) in a linear mixed‐effects model. Variance in fruit and seed residues was tested using a negative binomial generalized linear model with the function “glm.nb” from the package mass (Venables and Ripley 2002), with the covariates year and month, and ID as random factor.

A linear mixed‐effects model was conducted to explain the variation in BM of adult females and males. BM was corrected for body size (TL) and the covariates month (5 levels: May to September), *T*_aminhib_ (in° C), study site (3 levels), period (2 levels), and the interaction of month and period were included, ID was included as a random factor. To determine BM variance in the interactions and to receive an estimated marginal mean for all comparisons, we performed a Tukey post hoc test for all models with the “emmeans” and “contrast” functions in the package emmeans (Lenth 2021). To explain the variation in TL in adults, a linear mixed‐effects model was conducted with the covariates BM, period, sex, and month. For comparison among months, we performed a Tukey post hoc test.

To explain BM variation in juveniles, a log‐transformed linear mixed‐effects model was conducted with the covariates TL as a proxy for age, period, week of birth, and ID as a random factor. The residuals derived from each model were visually checked for variance homogeneity and normal distribution, and the data were transformed where necessary. The week of birth of juveniles was determined through a growth curve of one reference litter born on July 8, 2005, from which weekly measurements of TL were available until the age of 60 days. We modeled the growth of juvenile garden dormice using the Gompertz growth equation.

$$y\left(t\right)={\mathrm{ae}}^{\left(-{be}^{\left(-\mathrm{ct}\right)}\right)}$$

where $y\left(t\right)$ is the TL at the age (in days) at time t, a is the upper asymptote defined as the maximum TL, b is a scale parameter describing the exponential decrease, and c describe the growth rate over time in days. To fit the measured data on TL and age of juveniles to the model, we used the non‐linear least squares method with the function “nls” in R Studio (Posit team 2024). The goodness of fit of the model was obtained by calculating *R* ^2^. The age of the juveniles was calculated by solving the equation to TL. The week of birth was calculated by subtracting the age of the juveniles from the date of capture and converted to the week number of birth. To describe the reproduction pattern in the two periods, information on the birth date of each juvenile was included once in the analysis. To test whether the juveniles in both periods had the same TL (age) at first capture, a U‐test was performed. Statistical analyses were performed in R (R Core Team 2024) with the graphical user interface RStudio (Posit team 2024). Results were considered significant with *p < 0.05, **p < 0.01, or ***p < 0.001. Nonsignificant results are indicated by n.s., while results with *p* < 0.1 were marked as tendencies (.). In case of multiple comparisons between months, significance is indicated by different lowercase letters. *N* refers to the number of individuals, *n* refers to the number of captures.

## 3 Results

### 3.1 Dietary Composition

**FIGURE 1:**
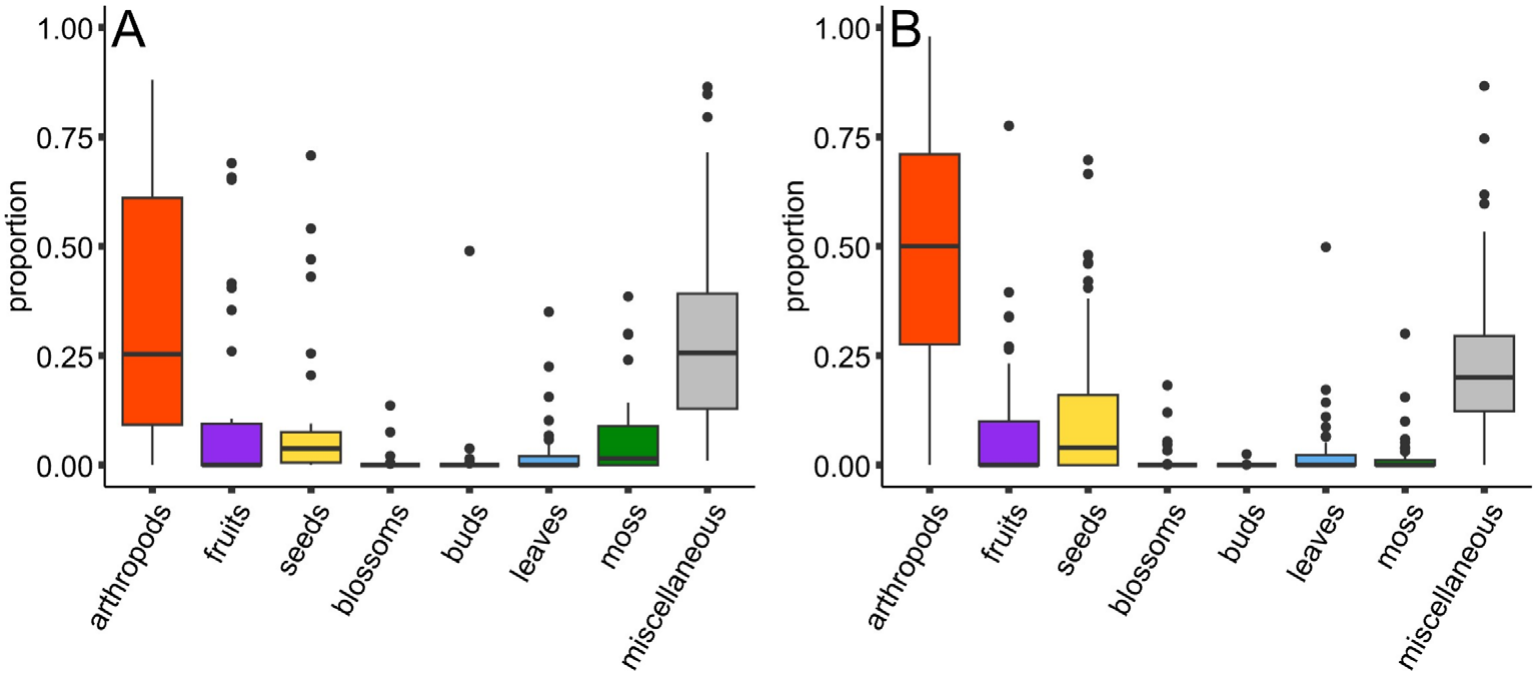
Proportions of different food residues in fecal samples of (A) adult (*n* = 31) and (B) juvenile (*n* = 47) garden dormice from May to September. The given values are median and quantile.

During the whole active season from May to September (2018–2019), in adults chitinous parts and whole arthropods accounted for 25% (median, *n* = 31, Figure 1A) of food residues whereas in juveniles they accounted for 50% (median, *n* = 47, Figure 1B). The proportions of residues of the other food categories in the fecal samples ranged between 0% and 9% (Figure 1).

### 3.2 Monthly Dietary Composition Adults

**FIGURE 2:**
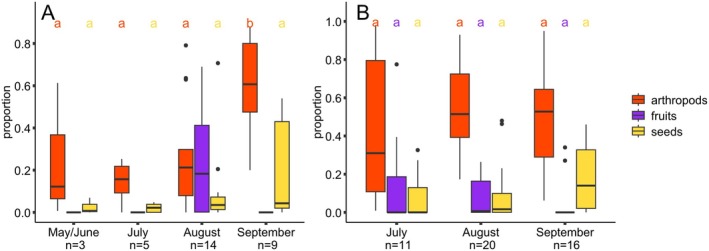
Seasonal change of the food residues of arthropods, fruits, and seeds in adult (A; *n* = 31) and juvenile garden dormice (B; *n* = 47). Different letters indicate statistical differences in the proportion of arthropod, fruit, and seed residues in fecal samples based on the linear mixed‐effect models (*p* < 0.05). The given values are median and quantile.

Arthropod residues were found in 80% to 100% of the adult fecal samples during the respective months. Seed residues were found in 67% to 88% of the samples collected, and fruits were found only in August in 71% of the samples (Table 4). The results of the linear mixed‐effect model show that the proportion of arthropod residues in the fecal samples of adults changed seasonally. During September, the proportion of arthropods was 61%, which was significantly higher than during the previous months (*n* = 9, Tukey post hoc test: *p* < 0.001 for all comparisons with September, Tables S1 and S2). The linear mixed‐effect model shows that the proportion of seed residues did not differ significantly between months (Tukey post hoc test: *p* > 0.31 for all comparisons). We found a tendency toward a lower proportion of seed residues in 2019 compared with 2018 (Tables S3 and S4). Fruit residues were only found in August with a median of 18% (*Q*_25_ = 0.2%, *Q*_75_ = 41%, *n* = 14, Figure 2A).

**TABLE 4:** Frequency of occurrence of the main food items arthropods, fruits, and seeds in the fecal samples (*n* = 87) of adult and juvenile garden dormice.

Adults	May/June (*n* = 3)	July (*n* = 5)	August (*n* = 14)	September (*n* = 9)
Arthropods	100%	80%	93%	100%
Fruits	0%	0%	71%	0%
Seeds	67%	60%	88%	89%

Juveniles		July (*n* = 11)	August (*n* = 20)	September (*n* = 16)
Arthropods	—	100%	100%	100%
Fruits	—	36%	55%	13%
Seeds	—	45%	65%	88%

### 3.3 Monthly Dietary Composition Juveniles

In juveniles, arthropods were found in all samples analyzed, seeds were found in 45% to 88% of the samples during the respective months, and the frequency of fruit residues in the fecal samples varied between 13% and 55% throughout the season (Table 4). Statistical analyses showed that the proportion of arthropod residues in the fecal samples of weaned juveniles was high throughout the whole season (median_July_ = 31%, *n* = 11; median_August_ = 51%, *n* = 20; median_September_ = 53%, *n* = 16) and did not change seasonally (Tukey post hoc test: *p* > 0.31 for all comparisons, Table S5). In 2018, the proportion of arthropod residues was 50% (SE ± 8%), which is significantly higher than in 2019 (34%, SE ± 8%, Table S6). The proportions of fruit residues were comparatively low throughout the season (median: 0%–0.65%) and did not change seasonally (Tukey post hoc test: *p* > 0.61 for all comparisons, Tables S7 and S8). The proportion of seeds ranged from 0% to 14% and did not show significant seasonal changes (Tukey post hoc test: *p* > 0.74 for all comparisons, Figure 2B, Tables S9 and S10).

### 3.4 BM Adults

**FIGURE 3:**
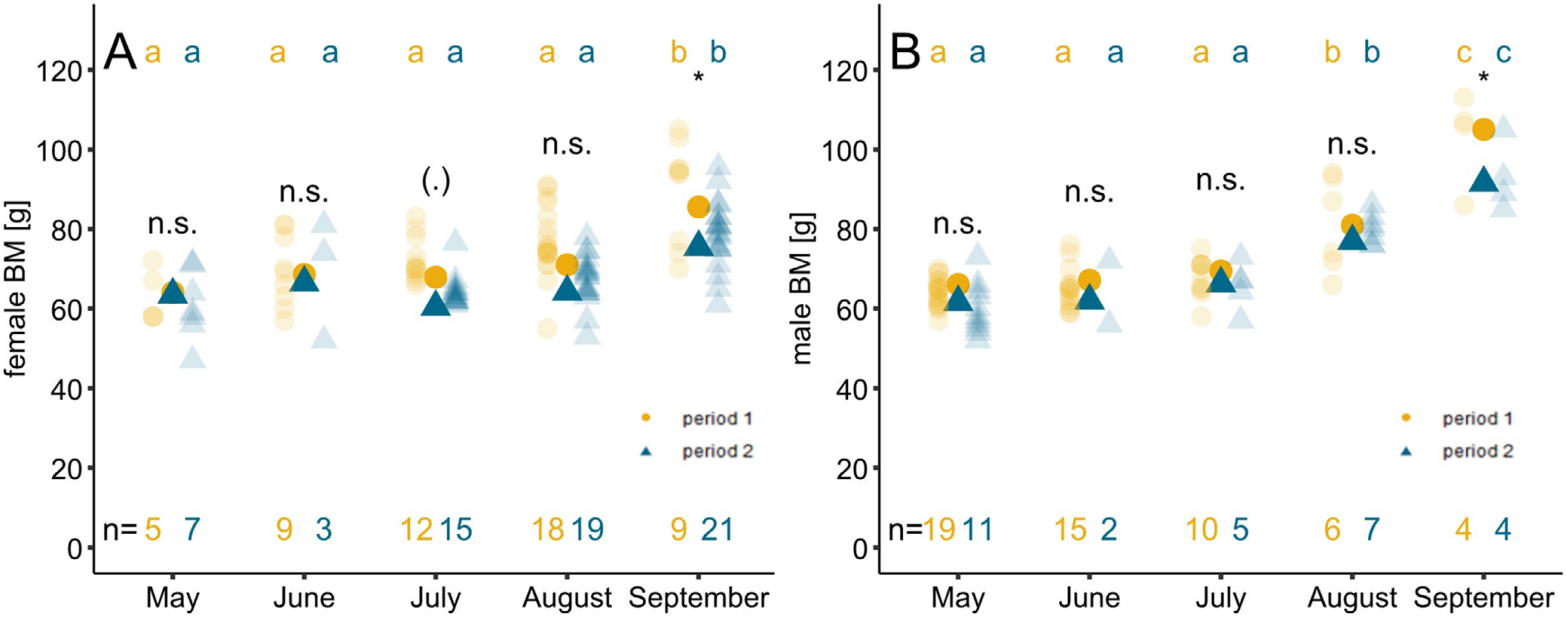
BM variation in adult females (A) and adult males (B) over the active season in the two periods. BM mean values derived from the model (yellow circles: Period 1 and blue triangles: Period 2). The shaded circles and triangles show the BM measured in the field for the different periods. Different lowercase letters indicate significant differences (*p* < 0.05) in BM between the months of the different time periods, divided into Period 1 (yellow) and Period 2 (blue). *n* refers to the sample size for the different periods. Significance levels are based on the results of Tukey post hoc tests.

After emergence from hibernation, female BM corrected for body size did not differ significantly between the two periods (May: mean_period1_ = 64 g, ±4.1 g, *n* = 5, mean_period2_ = 63.5 g, ±3.7 g, *n* = 7). However, females in Period 2 tended to have a lower BM in July compared with Period 1 (July: mean_period1_ = 67.9 g ±3.4 g, *n* = 12; mean_period2_ = 60.4 g ±3.2 g, *n* = 15). In both time periods, females increased their BM until September. However, BM in Period 2 during September was 10 g lower than in Period 1; this difference was significant (mean_period2_ = 75.5 g, SD ± 3 g, *n* = 21; mean_period1_ = 85.5 g, SD ± 3.6, *n* = 9, Figure 3A, Tables S11 and S12). BM was positively correlated with TL (*p* < 0.001), whereas *T*_aminhib_ and study site showed no significant correlations with BM (Tukey post hoc test_site_: *p* > 0.54 for all comparisons; Tables S13 and S14).

Male BM after emergence from hibernation corrected for body size did not differ between the two periods (May: mean_period1_ = 66.1 g, ±2.9 g, *n* = 19, mean_period2_ = 61.7 g, ±2.9 g, *n* = 11). In both time periods, males started pre‐hibernation fattening in August. However, shortly before entering hibernation in September, BM of adult males in Period 2 was significantly lower (91.8 g ± 4.1 g, *n* = 4) than in Period 1 (105.1 ± 4.2 g; *n* = 4, Figure 3B, Tables S15 and S16). TL positively correlated with BM (*p* = 0.08), *T*_aminhib_, and study site showed no significant correlation with BM (Tukey post hoc test_site_: *p* > 0.58 for all comparisons; Tables S17 and S18).

We found no difference in TL between the two periods. However, TL in males was 1.06 mm larger than in females (SE ± 0.29 mm), and TL was positively correlated with BM (Table 5). In yearlings, TL increased during the active season (Table S19).

**TABLE 5:** Results of the linear mixed‐effect model, explaining the variance in TL of adult garden dormice (*n* = 201, *N* = 61) reference level of factors: Month_May_, Period 1, sex_female_, variance of random effect: 0.81, residual variance: 0.85, $R_{m}^{2}$ = 0.25, $R_{c}^{2}$ = 0.61.

	Estimate	SE	df	*t*	*p*	
**(Intercept)**	**31.7**	**0.7**	**192.4**	**45.1**	< **0.001**	***
**BM**	**0.03**	**0.01**	**189**	**2.8**	< **0.01**	**
**Sex**_**male**_	**1.1**	**0.3**	**55.4**	**3.6**	< **0.001**	***
Period 2	0.01	0.3	52.2	0.03	0.98	n.s.
Month_June_	−0.09	0.3	177.7	−0.3	0.74	n.s.
**Month**_**July**_	**0.8**	**0.2**	**178.7**	**3.1**	< **0.01**	**
**Month**_**August**_	**1.3**	**0.3**	**171.1**	**4.8**	< **0.001**	***
Month_Sept_	0.6	0.4	178.7	1.7	< 0.1	(.)

*Note:* Significant differences are bold.

### 3.5 Juvenile Birth Date and Development

The Gompertz model was fitted to the growth data of the reference litter, resulting in estimated parameters for a with TL of 31.34 mm (SE ± 0.25 mm), scale parameter b with 1.22 (SE ± 0.03) and c being 0.07 (SE ± 0.003). The Gombertz model yielded a good fit to the data with an *R* ^*2*^ of 0.99 (Figure S1).

**FIGURE 4:**
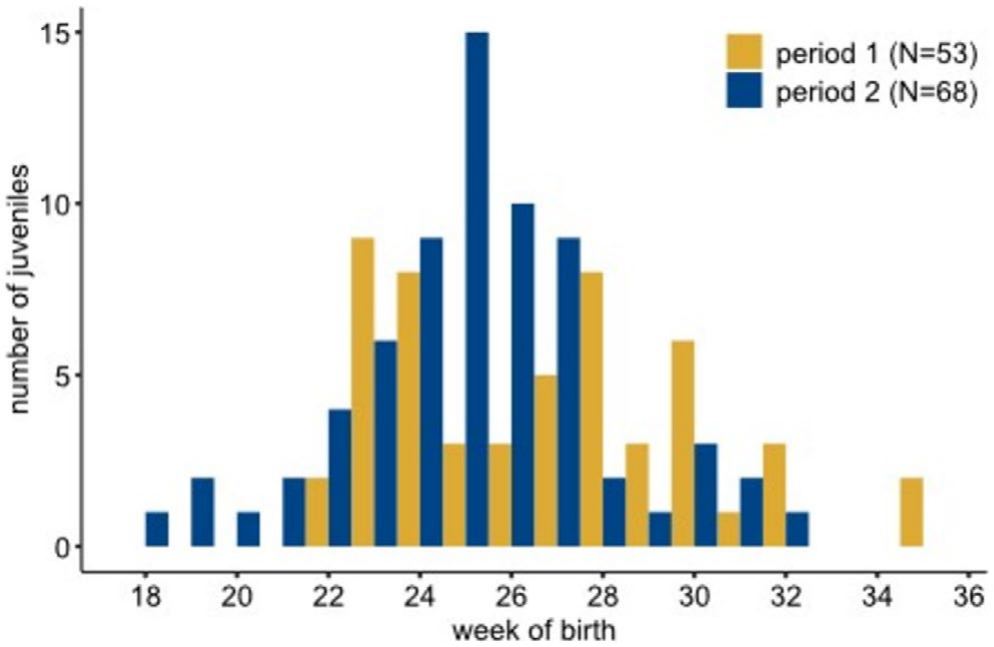
Histogram of the number of birth dates of juvenile garden dormice.

The week of birth of juvenile garden dormice could be determined in 121 individuals, 53 in Period 1 (26 females, 26 males, 1 sex unknown) and 68 in Period 2 (33 females, 35 males). In Period 1, the earliest births were calculated for Week 22, the latest births occurred at the end of August (Week 35), with two birth peaks in weeks 23 and 27. In Period 2, the earliest birth dates occurred in Week 18 (beginning of May) and the latest births were calculated for Week 32, with a peak in the number of births in Week 25 (Figure 4). TL (age) of first captured juveniles did not differ between the two periods (U‐test: *W* = 1983, *p* = 0.35).

**FIGURE 5:**
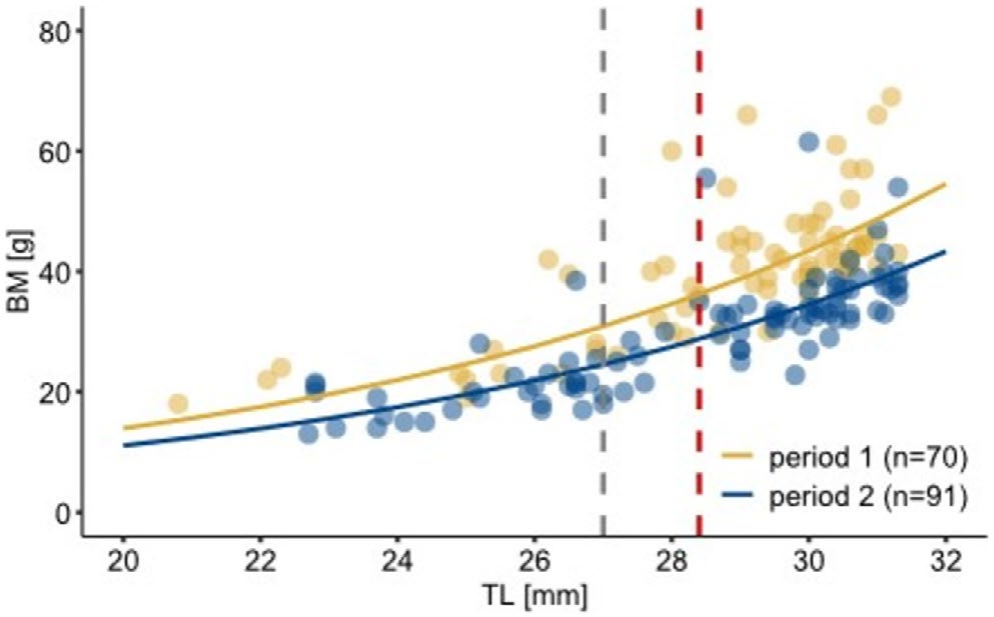
Estimated BM (line) for different TL of juvenile garden dormice during the two periods, divided into Period 1 (yellow) and Period 2 (blue). The shaded circles represent the original data measured in the field. The gray dashed line indicates the age of 29 days when juveniles leave their nests, and the red dashed line indicates weaning at the age of 34 days. Note that for better interpretation, the estimated values of the model were back transformed.

In both periods, juvenile BM was positively correlated with TL. The analysis revealed that in Period 2, BM increase was lower compared with Period 1 (Figure 5). The week of birth was positively correlated with BM, showing that later‐born juveniles gained BM faster (Table 6).

**TABLE 6:** Results of the log‐transformed linear mixed‐effect model, explaining the variance in BM of juveniles (*n* = 161, *N* = 121). $R_{m}^{2}$ = 0.80, $R_{c}^{2}$ = 0.80. Reference level of the factor period is Period 1.

	Estimate	SE	df	*t*	*p*	
(Intercept)	−0.32	0.20	157	−1.61	0.11	n.s.
**TL**	**0.11**	**0.005**	**157**	**21.56**	< **0.001**	***
**Week of birth**	**0.03**	**0.004**	**157**	**6.63**	< **0.001**	***
**Period 2**	**−0.23**	**0.03**	**157**	**−8.77**	< **0.001**	***

*Note:* Significant values are bold.

## 4 Discussion

In this study, we found that in Period 2 pre‐hibernation BM of garden dormice, corrected for body size, was 12% (10 g) lower than it used to be two decades ago. TL, as a proxy for body size, showed no change over time. During the same time period, juveniles showed a diminished BM gain after weaning. Individual BM variation depends on the availability of food resources and energy expenditure (Bright Ross et al. 2021; Korslund and Steen 2006). As an omnivorous species, garden dormice feed on a large variety of food resources (Gil‐Delgado et al. 2010; Kuipers et al. 2012; Dooren et al. 2024). Our results indicate that arthropods represented an important food resource for garden dormice in the Black Forest, as arthropod residues were found in almost all fecal samples and accounted for large proportions in the samples. This was especially true during juvenile growth and pre‐hibernation fattening, where all samples contained arthropod residues and were found in relatively high proportions. Seeds and berries (e.g., blueberries, blackberries) were found in lower frequencies within the fecal samples. However, the seasonal comparison of all food residues with May/June must be taken with caution, as we only had a sample size of 3. Mammals are known to actively select food resources to meet their specific dietary requirements (Diedrich et al. 2014; Giroud et al. 2018; Hertel et al. 2018; Remonti et al. 2016). Based on our findings, we assume that garden dormice depend on arthropods as a high‐quality food resource during pre‐hibernation fattening and juvenile growth. A high amount of insects in the diet of garden dormice was also found in other studies (Gil‐Delgado et al. 2010; Kuipers et al. 2012, Dooren et al. 2024). However, as the digestibility of food items differs, our semi‐quantitative approach to dietary analysis is likely to have led to some inaccuracy in estimating the proportions of different food residues. For example, garden dormice are known to consume earthworms and snails (Kuipers et al. 2012; Dooren et al. 2024), of which the few remains are hard to detect in the feces. Also in fruits, the actual proportion may be under‐ or overestimated depending on the relative size of their seeds and whether the seeds are swallowed or not. It is therefore likely that the proportion of arthropods is overestimated as the chitinous exoskeletons are indigestible (Dickman and Huang 1988; Matthews et al. 2020). However, in our study, arthropods were found in almost all fecal samples analyzed, which strongly suggests that arthropods represent the primary food resource consumed during pre‐hibernation fattening and juvenile growth.

As garden dormice are fat‐storing hibernators that stop feeding during hibernation and rely completely on their body fat resources, reduced pre‐hibernation BM can negatively influence winter survival (Jebb et al. 2021; Speakman and Racey 1989). If energy expenditure during hibernation exceeded accumulated energy reserves, hibernators may not survive hibernation, have to terminate hibernation earlier when food resources are still scarce, and/or emerge with a reduced BM (Fietz et al. 2020; Humphries et al. 2003; Turbill and Prior 2016). In our study, males lost 39 g of their BM, that is, 0.18 g (0.17%) BM per day during a hibernation period which lasts from October until April in Period 1 (Tables S15 and S16). This loss is comparable to the findings in the laboratory, where dormice decreased their BM by 0.2%–0.26% per day hibernating at a *T*_a_ of 4.2°C starting with a pre‐hibernation BM of 142 g (Pajunen 1970, 1974). In Period 2, dormice started hibernation with a BM 10 g less than they used to 20 years ago. This means that their energy resources should have been used up 54 days earlier and they should have emerged already in March. As we did not check nest boxes as early as March, we do not know whether dormice in Period 2 have indeed emerged that early. However, garden dormice sightings in Germany are frequently reported by citizen scientists, with the earliest sighting in the Black Forest reported in March 2019–2024 (https://meldestelle.gartenschlaefer.de), and garden dormice were found in mid‐April in bird nest boxes regularly checked at high altitudes in the Black Forest National Park (M.I. F. pers. observation). From our capture‐mark‐recapture study, we have no proof that garden dormice extended their active season in autumn. In the study area, the mean daily *T*_a_ in March in Period 2 was 8.8°C with a mean daily minimum *T*_a_ around 4.7°C (SE ± 6.3°C). Food resources are scarce at this time of the year, and snow occurs until April. Thus, we assume that dormice emerging in March or April would not be able to survive.

We found no difference in BM corrected for body size after hibernation in May between the two periods. A possible explanation is that in Period 2, only those individuals survived which had a BM at the upper range before hibernation. Alternatively, garden dormice could adapt their hibernation physiology to their body fat reserves by reducing the frequency of arousals. Arousals are short returns to euthermia during which hibernators actively increase their *T*_b_. Arousals account for at least 70% of the energy consumed during hibernation and are associated with increased oxidative stress (Carey et al. 2000; Giroud et al. 2023; Karpovich et al. 2009; Wang 1979). Bieber et al. (2014) showed for edible dormice that heavier individuals showed more frequent arousals, presumably avoiding the negative effects of torpor, such as memory loss (Millesi et al. 2001), impaired immune response (Prendergast et al. 2002), or inhibited gonad maturation (Barnes et al. 1986). Potentially, garden dormice with a lower pre‐hibernation BM may exhibit arousals less frequently during hibernation, with a trade‐off between negative effects of torpor and oxidative stress of arousals.

The increase of arthropods as a food resource used during pre‐hibernation fattening indicates that garden dormice may actively switch their diet to be prepared for hibernation. Insects and other arthropods are rich in protein and fat (Oonincx and Finke 2021). For hibernators, access to a diet rich in unsaturated fatty acids is of enormous importance. In particular, mono‐ and polyunsaturated fatty acids (MUFAs, PUFAs) consumed and stored in white adipose tissue influence the ability to enter torpor, tolerated *T*_bmin_, torpor bout duration, and consequently energy consumed during hibernation (Florant et al. 1993; Geiser et al. 1994; Ruf and Arnold 2008). The required amount and composition of fatty acids vary between hibernating species. As PUFAs cannot be synthesized by mammals, they have to be consumed through their diet, and hibernators have been shown to optimize hibernation performance by changing their diet and their fatty acid intake (Diedrich et al. 2014; Frank et al. 1998). For instance, the insectivorous bat *Rhinopoma microphyllum* changes its diet during pre‐hibernation fattening from beetles and bugs to queen carpenter ants ( *Camponotus fellah* ), which are rich in saturated and monounsaturated fatty acids (Levin et al. 2013). In a laboratory study, golden‐mantled ground squirrels ( *Callospermophilus lateralis* ) actively chose the PUFA content in their diet to meet an optimal level for their hibernation performance (Frank 1992). Similarly, we assume that garden dormice may actively switch to arthropods as a main diet to optimize their hibernation performance.

Juveniles living in a seasonal environment have to grow rapidly and accumulate sufficient energy reserves to survive upcoming unfavorable environmental conditions, such as winter. This is particularly important for hibernators, as juveniles must not only consume sufficient energy and protein for their growth but also have to accumulate body fat to survive their first hibernation period (Lenihan and Van Vuren 1996; Rieger 1996). In line with the findings of our study, Stumpfel et al. (2017) showed that in captivity late‐born juveniles exhibited a faster growth rate compared with earlyborn juveniles. However, late‐born juveniles showed a lower pre‐hibernation body fat content than early born juveniles (Mahlert et al. 2018). In male European ground squirrels ( *Spermophilus citellus* ), protein‐supplemented juveniles had an enhanced growth rate compared with those fed with a standard diet or a diet enriched with PUFAs (Strauss et al. 2007). Furthermore, deer mice ( *Peromyscus maniculatus borealis* ) supplemented with protein showed higher juvenile growth rates and earlier reproductive maturity (McAdam and Millar 1999). Juvenile European hedgehogs ( *Erinaceus europaeus* ) need to weigh at least 450 g to survive the next hibernation period (Morris 1984) and hazel dormice ( *Muscardinus avellanarius* ) are assumed to require a critical pre‐hibernation BM of more than 15 g (Csorba 2003). In our study, we found that juvenile garden dormice in Period 2 had a diminished BM increase compared with juveniles in Period 1. This can be seen as an early warning signal, as reduced BM likely affects the chance of surviving the upcoming winter (Ronget et al. 2018). At the same time, for juvenile garden dormice of this study, arthropods represented the main food resource after weaning and during pre‐hibernation fattening. As insects are rich in protein, they represent a high‐quality resource crucial for promoting juvenile growth (Oonincx and Finke 2021; Woods and Armitage 2003). We assume that juvenile garden dormice in Period 2 were unable to acquire sufficient arthropod food and therefore showed a slower increase in BM than in Period 1. We acknowledge that our sample sizes in the BM analyses are small in some months, which may limit our statistical power. However, the effects found throughout this study are consistent across both age classes and sexes. Therefore, we believe that our conclusions are robust despite the small sample size.

In addition to BM changes in adults and juveniles, we observed unexpected changes in the pattern of birth dates. While we could detect only one peak of births in Period 2, we observed two birth peaks in Period 1. Consistent with this pattern, genetic maternity analysis revealed that in Period 2 only one of the nine females gave birth to two litters within one year (Erhardt et al. 2024). This change in the reproduction pattern could also be explained by changes in food availability, thereby limiting the energy available for reproduction and preventing females from reproducing (Fietz et al. 2005; Ruf et al. 2006). In seed‐eating rodents, reproduction is positively correlated with seed availability (Wolff 1996). Low food availability can also prevent reproducing females from compensating for the high energetic costs during lactation (Speakman 2008). Females in our study had a tendency toward a lower BM after lactation in July in Period 2 compared with Period 1. Therefore, the potential reduction in the number of litters, together with the tendency to lower BM in females after lactation, could serve as early warning signals for future population decline (Cerini et al. 2023).

Reasons for the changes in pre‐hibernation BM, slower juvenile growth, and reproduction pattern over the last two decades can be manifold. For example, climate change has been shown to have far‐reaching consequences for hibernators like the garden dormouse. Because of increasing winter temperatures, hibernators may have elevated energetic costs during hibernation (Humphries et al. 2002), changing their BM at emergence and/or their phenology (e.g., Fietz et al. 2020; Inouye et al. 2000; Wells et al. 2022). However, in our study, we did not find a shift in the mean birth date between the two time periods. As garden dormice start reproducing directly after emergence, the birth date can be used as a proxy for the date of emergence (Millesi et al. 1999). Different from the edible dormouse, we did not find an effect of *T*_aminhib_ on BM after emergence in adult garden dormice (Fietz et al. 2020). Thus, warm winters do not seem to affect hibernation phenology and BM at emergence in this species at our study site so far. This may be because of the circumstance that here, at an altitude of about 900 m a.s.l., winter temperatures are still within the thermal optimum for hibernating garden dormice (Pajunen 1979). However, the ongoing climate change will further increase winter temperatures even at higher altitudes (IPCC 2018), which might possibly reduce winter survival probabilities of this population.

Another explanation for our results is a decline in food availability (Bright Ross et al. 2021; Grames et al. 2023; Hall et al. 2018). Although speculative, the lower pre‐hibernation BM observed in adults, the slower juvenile growth, and the change in reproduction pattern could be consequences of the ongoing insect decline (Hallmann et al. 2017; Seibold et al. 2019; Wagner 2020). Insects are important for ecosystem functioning; the loss in abundance, biomass, and diversity is a major threat for biodiversity and human wellbeing worldwide (Sánchez‐Bayo and Wyckhuys 2019; Wagner 2020). To date, there are only a few studies concerning the effects of insect decline on higher trophic levels, with most of them focusing on birds (Bowler et al. 2019; Spiller and Dettmers 2019; Tallamy and Shriver 2021). For example, Møller (2019) was able to confirm a relationship between the decline in flying insects killed on windscreens of cars, the abundance of breeding barn swallows ( *Hirundo rustica* ), and the rate of food provisioning to their offspring. Similarly, it was shown that the abundance of insectivorous birds is associated with the abundance of insects (Møller et al. 2021), and Martay et al. (2023) showed that the fledgling survival of barn swallows is positively correlated with insect biomass. A recent meta‐analysis on songbirds found a positive correlation between invertebrate food availability, offspring body condition, and reproductive success (Grames et al. 2023). For the tropical Luquillo rainforest of Puerto Rico, Lister and Garcia (2018) reported a decrease in abundance of arthropods between 1976 and 2012, possibly caused by elevated *T*_a_, which was associated with a decrease in insectivorous lizards, frogs, and birds.

Therefore, we believe that for garden dormice, the limitation of insect food resources may cause a bottom‐up limitation that affects survival and reproductive success and thus population density (Hertel et al. 2018; Prevedello et al. 2013). The decline of pre‐hibernation and juvenile BM, as well as the change in reproduction pattern, can be considered aearly warning signals preceding population decline. This is consistent with our finding that no garden dormice were captured in Period 2 at the ME study site, indicating that they no longer occur there. These findings are even more worrying as we were studying a population of garden dormice that is regarded to be thriving and seems so far unaffected by the dramatic decline of this species (Büchner et al. 2024). However, even though our study sites were located within a strictly protected area (since 2014) in which the use of pesticides is prohibited, it is likely that pesticide pollution can affect local insect populations. Unpublished data from a pesticide sampler installed for several years by the National Park administration on the Schliffkopf summit, far away from any agricultural areas, show that numerous pesticides are regularly detectable even at a greater distance from the site of application and can therefore naturally also affect insect diversity and abundance (M.I.F. pers. observation). A recently published study showed that spraying pesticides in orchards can cause contamination even in protected areas and areas remote from the application sites (Brühl et al. 2024). Although our study highlights the potential impacts of insect decline on the omnivorous garden dormouse population, more research is needed to understand the impact of insect decline on higher trophic levels and to develop conservation strategies that mitigate these cascading effects within ecosystems.

## Author Contributions

**Stefanie Erhardt:** conceptualization (equal), data curation (lead), formal analysis (lead), funding acquisition (equal), investigation (lead), methodology (equal), resources (equal), visualization (lead), writing – original draft (lead). **Marc I. Förschler:** conceptualization (equal), methodology (equal), project administration (supporting), resources (supporting), writing – review and editing (equal). **Joanna Fietz:** conceptualization (equal), formal analysis (supporting), funding acquisition (equal), investigation (supporting), methodology (equal), project administration (lead), resources (equal), supervision (lead), writing – review and editing (equal).

## Conflicts of Interest

The authors declare no conflicts of interest.

## Supporting information


Appendix S1.

## Data Availability

Data are available at Figshare https://doi.org/10.6084/m9.figshare.27960402.v2.
