# Supplementary material for: Reduced Body Mass in a Highly Insectivorous Mammal, the Garden Dormouse—Ecological Consequences of Insect Decline?
Source: Ecol Evol. 2025 Apr 21;15(4):e71340. doi: 10.1002/ece3.71340 (PMC12011555; doi:10.1002/ece3.71340)
Supplement: Supplementary file 1 — Appendix S1. [file ECE3-15-e71340-s001.docx]

**Supporting Information**

Reduced body mass in a highly insectivorous mammal, the garden dormouse - Ecological consequences of insect decline?

Stefanie Erhardt^1,2^, Marc I. Förschler^3^, Joanna Fietz^1,2^

^1^ University of Hohenheim, Institute of Biology, Department of Zoology, Stuttgart, Germany

^2^ KomBioTa – Center of Biodiversity and Integrative Taxonomy, University of Hohenheim, Stuttgart, Germany

^3^ Black Forest National Park, Department for Ecological Monitoring, Research and Species Protection, Seebach, Germany

Corresponding author: Stefanie.erhardt@uni-hohenheim.de

Table S1: Results of the linear mixed effect model explaining the variance of the proportion of arthropod residues in the fecal samples of adult garden dormice (n = 31, N = 15). Reference level is the month_May/June_, sex_female_, year_2018_. Random factor: ID, variance of random effect is 0.08, residual variance is 0.01, R^2^m = 0.39, R^2^c = 0.91.

|  | estimate | SE | df | t | p |  |
| --- | --- | --- | --- | --- | --- | --- |
| (Intercept) | 0.21 | 0.15 | 25 | 1.45 | 0.16 | n.s. |
| month_July_ | 0.08 | 0.1 | 13.02 | 0.74 | 0.47 | n.s. |
| month_August_ | 0.11 | 0.1 | 14.08 | 1.05 | 0.31 | n.s. |
| **month_Sep._** | **0.47** | **0.12** | **15.38** | **3.91** | **< 0.01** | ****** |
| year_2019_ | -0.2 | 0.11 | 23.31 | -1.73 | 0.09 | (.) |
| sex_male_ | 0.21 | 0.18 | 13.12 | 1.17 | 0.26 | n.s. |

Table S2: Tukey post-hoc test for the factor month of the linear mixed effect model explaining the variance in arthropod residuals in fecal samples of adult garden dormice (n = 31, N = 15).

|  | estimate | SE | z | p |  |
| --- | --- | --- | --- | --- | --- |
| month_July_ – month_May/June_ | 0.08 | 0.1 | 0.74 | 0.88 | n.s. |
| month_August_ – month_May/June_ | 0.11 | 0.1 | 1.05 | 0.71 | n.s. |
| **month_Sep._ – month_May/June_** | **0.47** | **0.12** | **3.91** | **<0.001** | ******* |
| month_August_ – month_July_ | 0.03 | 0.07 | 0.44 | 0.97 | n.s. |
| **month_Sept._ – month_July_** | **0.4** | **0.09** | **4.43** | **<0.001** | ******* |
| **month_Sep._ – month_August_** | **0.37** | **0.08** | **4.52** | **<0.001** | ******* |

Table S3: Results of the linear mixed effect model explaining the square root transformed variance of the proportion of seed residues in the fecal samples of adult garden dormice (n = 31, N = 15). Reference level is the month May/June. Random factor = ID, variance of random effect is 0.02, residual variance is 0.03, R^2^m = 0.26, R^2^c = 0.53.

|  | estimate | SE | df | t | p |  |
| --- | --- | --- | --- | --- | --- | --- |
| (Intercept) | 0.28 | 0.14 | 22.45 | 1.98 | 0.06 | (.) |
| month_July_ | -0.02 | 0.14 | 13.05 | -0.17 | 0.87 | n.s. |
| month_August_ | 0.14 | 0.13 | 20.63 | 1.1 | 0.28 | n.s. |
| month_Sep._ | 0.11 | 0.15 | 23.43 | 0.76 | 0.45 | n.s. |
| year_2019_ | -0.2 | 0.11 | 13.35 | -1.9 | 0.08 | (.) |
| sex_male_ | -0.12 | 0.13 | 7.44 | -0.95 | 0.37 | n.s. |

Table S4: Tukey post-hoc test for the factor month of the linear mixed effect model explaining the variance in seed residuals in fecal samples of adult garden dormice (n = 31, N = 15).

|  | estimate | SE | z | p |  |
| --- | --- | --- | --- | --- | --- |
| month_July_ – month_May/June_ | -0.02 | 0.14 | -0.17 | 0.998 | n.s. |
| month_August_ – month_May/June_ | 0.14 | 0.13 | 1.1 | 0.68 | n.s. |
| month_Sep._ – month_May/June_ | 0.11 | 0.15 | 0.76 | 0.89 | n.s. |
| month_August_ – month_July_ | 0.17 | 0.1 | 1.70 | 0.32 | n.s. |
| month_Sept._ – month_July_ | 0.14 | 0.12 | 1.12 | 0.64 | n.s. |
| month_Sep._ – month_August_ | -0.03 | 0.1 | -0.29 | 0.99 | n.s. |

Table S5: Tukey post-hoc test for the factor month of the linear mixed effect model explaining the variance in the proportion of arthropod residuals in fecal samples of juvenile garden dormice (n = 47, N = 33).

|  | estimate | | SE | z | p |  |
| --- | --- | --- | --- | --- | --- | --- |
| month_August_ – month_July_ | 0.15 | 0.1 | | 1.46 | 0.31 | n.s. |
| month_Sept._ – month_July_ | 0.06 | 0.1 | | 0.63 | 0.81 | n.s. |
| month_Sep._ – month_August_ | -0.08 | 0.09 | | -0.94 | 0.62 | n.s. |

Table S6: Results of the linear mixed effect model explaining the variance of the proportion of arthropod residuals in fecal samples in juvenile garden dormice (n=47, N=35). Reference level of the factor: month_July_, year_2018_. Random factor = ID, R^2^m = 0.10.

|  | estimate | SE | df | t | p |  |
| --- | --- | --- | --- | --- | --- | --- |
| **(Intercept)** | **0.5** | **0.08** | **43** | **6.07** | **< 0.001** | ******* |
| month_August_ | 0.15 | 0.01 | 43 | 1.46 | 0.15 | n.s. |
| month_Sep._ | 0.06 | 0.1 | 43 | 0.63 | 0.54 | n.s. |
| **year_2019_** | **-0.16** | **0.08** | **43** | **-2.06** | **< 0.05** | ***** |

Table S7: Results of the negative binomial generalized linear model explaining the variance of the proportion of fruit residuals in fecal samples in juvenile garden dormice (n = 47, N = 33). Reference level of the factor: month_July_, year_2018_. Random factor = ID, R^2^m = 0.09.

|  | estimate | SE | z | p |  |
| --- | --- | --- | --- | --- | --- |
| **(Intercept)** | **-2.0** | **0.84** | **-2.37** | **< 0.05** | ***** |
| month_August_ | -0.58 | 1.11 | -0.52 | 0.61 | n.s. |
| month_Sep._ | -1.31 | 1.37 | -0.95 | 0.34 | n.s. |
| year_2019_ | 0.1 | 1.04 | 0.1 | 0.92 | n.s. |

Table S8: Tukey post-hoc test for the factor month of the linear mixed effect model explaining the variance in the proportion of fruit residuals in fecal samples of juvenile garden dormice.

|  | estimate | SE | z | p |  |
| --- | --- | --- | --- | --- | --- |
| month_August_ – month_July_ | -0.58 | 1.11 | -0.52 | 0.86 | n.s. |
| month_Sept._ – month_July_ | -1.31 | 1.37 | -0.95 | 0.61 | n.s. |
| month_Sep._ – month_August_ | -0.73 | 1.45 | -0.5 | 0.87 | n.s |

Table S9: Results of the negative binomial generalized linear model explaining the variance of the proportion of seed residuals in fecal samples in juvenile garden dormice (n = 47, N = 33). Reference level of the factor: month_July_, year_2018_. Random factor = ID, R^2^m = 0.11.

|  | estimate | SE | z | p |  |
| --- | --- | --- | --- | --- | --- |
| **(Intercept)** | **-2.27** | **0.98** | **-2.34** | **0.02** | ***** |
| month_August_ | 0.31 | 1.27 | 0.24 | 0.81 | n.s. |
| month_Sep._ | 0.81 | 1.1 | 0.74 | 0.45 | n.s. |
| year_2019_ | -0.85 | 0.96 | -0.9 | 0.37 | n.s. |

Table S10: Tukey post-hoc test for the factor month of the linear mixed effect model explaining the variance in the proportion of seed residuals in fecal samples of juvenile garden dormice.

|  | estimate | SE | z | p |  |
| --- | --- | --- | --- | --- | --- |
| month_August_ – month_July_ | 0.31 | 1.27 | 0.24 | 0.97 | n.s. |
| month_Sept._ – month_July_ | 0.81 | 1.1 | 0.74 | 0.74 | n.s. |
| month_Sep._ – month_August_ | 0.5 | 1 | 0.5 | 0.87 | n.s. |

Table S11: Estimated marginal means for the interaction month x period of the linear mixed effect model explaining the variance in BM of female garden dormice.

| month | period | emmean | SE | df | lower CI | upperCI |
| --- | --- | --- | --- | --- | --- | --- |
| May | *period 1* | 64 | 4.1 | 73.6 | 55.7 | 72.3 |
| June | *period 1* | 68.5 | 3.5 | 49.6 | 61.5 | 75.6 |
| July | *period 1* | 67.9 | 3.4 | 46.7 | 61.1 | 74.7 |
| August | *period 1* | 71 | 3.2 | 38.1 | 64.5 | 77.5 |
| Sep. | *period 1* | 85.5 | 3.6 | 55 | 78.3 | 92.8 |
| May | *period 2* | 63.5 | 3.7 | 63.1 | 56.2 | 70.8 |
| June | *period 2* | 66.5 | 4.9 | 93 | 56.9 | 76.2 |
| July | *period 2* | 60.4 | 3.2 | 41 | 54 | 66.9 |
| August | *period 2* | 64.2 | 3 | 36.5 | 58.1 | 70.3 |
| Sep. | *period 2* | 75.5 | 3 | 34.6 | 69.4 | 81.5 |

Table S12: Post-hoc test for the interaction month x period of the linear mixed effect model explaining the variance in BM of female garden dormice.

|  | *contrast* | *estimate* | *SE* | *df* | *lowerCI* | *upperCI* | *t* | *p* |  |
| --- | --- | --- | --- | --- | --- | --- | --- | --- | --- |
| *period 1* | *June-May* | 4.5 | 3.8 | 93.2 | -3.0 | 12.1 | 1.12 | 0.23 | n.s. |
| *period 1* | *July-May* | 3.9 | 3.9 | 96.7 | -3.8 | 11.6 | 1.0 | 0.32 | n.s. |
| *period 1* | *July-June* | -0.7 | 3.0 | 94.3 | -6.6 | 5.3 | -0.2 | 0.82 | n.s. |
| *period 1* | *August-May* | 7.0 | 4.0 | 97.9 | -0.9 | 15.0 | 1.8 | 0.08 | n.s. |
| *period 1* | *August-June* | 2.5 | 3.0 | 100.1 | -3.5 | 8.5 | 0.8 | 0.42 | n.s. |
| *period 1* | *August-July* | 3.1 | 2.4 | 89.4 | -1.7 | 8.0 | 1.3 | 0.2 | n.s. |
| *period 1* | ***Sep.-May*** | **21.5** | **4.4** | **97.8** | **12.8** | **30.3** | **4.9** | **< 0.001** | ******* |
| *period 1* | ***Sep.-June*** | **17.0** | **3.6** | **100.4** | **9.9** | **24.1** | **4.8** | **< 0.001** | ******* |
| *period 1* | ***Sep.-July*** | **17.7** | **3.0** | **92.9** | **11.7** | **23.6** | **5.9** | **< 0.001** | ******* |
| *period 1* | ***Sep.-August*** | **14.5** | **2.6** | **89** | **9.4** | **19.7** | **5.6** | **< 0.001** | ******* |
| *period 2* | *June-May* | 3.0 | 4.8 | 93.3 | -6.5 | 12.6 | 0.6 | 0.53 | n.s. |
| *period 2* | *July-May* | -3.1 | 3.2 | 94.3 | -9.4 | 3.2 | -1.0 | 0.33 | n.s. |
| *period 2* | *July-June* | -6.1 | 4.8 | 99.4 | -15.6 | 3.4 | -1.3 | 0.2 | n.s. |
| *period 2* | *August-May* | 0.7 | 2.9 | 86 | -5.0 | 6.5 | 0.3 | 0.8 | n.s. |
| *period 2* | *August-June* | -2.3 | 4.4 | 96.8 | -11.0 | 6.4 | -0.5 | 0.6 | n.s. |
| *period 2* | *August-July* | 3.8 | 2.4 | 92.6 | -0.9 | 8.6 | 1.6 | 0.11 | n.s. |
| *period 2* | ***Sep.-May*** | **12.0** | **2.9** | **88.9** | **6.2** | **17.8** | **4.1** | **< 0.001** | ******* |
| *period 2* | *Sep.-June* | 8.9 | 4.6 | 97.7 | -0.1 | 18.0 | 2.0 | 0.053 | (.) |
| *period 2* | ***Sep.-July*** | **15.1** | **2.3** | **96** | **10.5** | **19.6** | **6.5** | **< 0.001** | ******* |
| *period 2* | ***Sep.-August*** | **11.2** | **2.0** | **84.6** | **7.2** | **15.3** | **5.6** | **< 0.001** | ******* |
| *month_May_* | *period 2-period 1* | -0.5 | 5.5 | 79 | -11.5 | 10.5 | -0.1 | 0.93 | n.s. |
| *month_June_* | *period 2-period 1* | -2.0 | 5.8 | 90.9 | -13.6 | 9.5 | -0.3 | 0.73 | n.s. |
| *month_July_* | *period 2-period 1* | -7.5 | 4.4 | 50.4 | -16.2 | 1.3 | -1.7 | 0.09 | (.) |
| *month_August_* | *period 2-period 1* | -6.8 | 4.2 | 44.1 | -15.2 | 1.7 | -1.6 | 0.11 | n.s. |
| *month_Sep._* | ***period 2-period 1*** | **-10.1** | **4.4** | **51.1** | **-18.9** | **-1.3** | **-2.3** | **< 0.05** | ***** |

Table S13: Results of the linear mixed effect model, explaining the variance in BM of female garden dormice (n = 118, N = 29) reference level of factors: month_May_, time period_2003-2005_, site_ME,_, random factor = ID. Variance of random effect: 43.8, residual variance: 34.5, R^2^m = 0.48, R^2^c = 0.72. Significant differences are bold.

|  | estimate | SE | df | t | p |  |
| --- | --- | --- | --- | --- | --- | --- |
| (Intercept) | -27.4 | 23.5 | 94.9 | -1.2 | 0.25 | n.s. |
| month_June_ | 4.5 | 3.8 | 90.0 | 1.2 | 0.23 | n.s. |
| month_July_ | 3.9 | 3.8 | 94.4 | 1.0 | 0.32 | n.s. |
| month_August_ | 7.0 | 4.0 | 95.9 | 1.8 | 0.08 | (.) |
| **month_Sep._** | **21.5** | **4.4** | **95.8** | **4.9** | **< 0.001** | ******* |
| **TL** | **2.6** | **0.7** | **99.5** | **3.7** | **< 0.001** | ******* |
| period_2_ | -0.5 | 5.5 | 73.0 | -0.1 | 0.93 | n.s. |
| site_RL_ | 5.6 | 5.4 | 20.9 | 1.0 | 0.31 | n.s. |
| site_WK_ | 3.56 | 7.1 | 19.3 | 0.5 | 0.62 | n.s. |
| T_aminhib_ | 0.2 | 3.5 | 74.3 | 0.05 | 0.96 | n.s. |
| month_June_: period 2 | -1.5 | 6.1 | 91.7 | -0.2 | 0.80 | n.s. |
| month_July_: period 2 | -7.0 | 4.8 | 92.0 | -1.5 | 0.15 | n.s. |
| month_August_: period 2 | -6.3 | 4.6 | 90.7 | -1.4 | 0.18 | n.s. |
| month_Sep._: period 2 | -9.6 | 4.9 | 92.5 | -2.0 | 0.054 | (.) |

Table S14: Tukey post-hoc test for the factor study site of the linear mixed effect model explaining the variance in BM of female garden dormice.

|  | estimate | SE | z | p |  |
| --- | --- | --- | --- | --- | --- |
| site_RL_ – site_ME_ | 5.6 | 5.4 | 1.0 | 0.54 | n.s. |
| site_WK_ – site_ME_ | 3.6 | 7.1 | 0.5 | 0.86 | n.s. |
| site_WK_ – site_RL_ | -2.1 | 4.5 | -0.5 | 0.88 | n.s. |

Table S15: Estimated marginal means for the interaction month x period of the linear mixed effect model explaining the variance in BM of male garden dormice.

| *month* | *period* | *emmean* | *SE* | *df* | *lower CI* | *upperCI* |
| --- | --- | --- | --- | --- | --- | --- |
| *May* | *period 1* | 66.1 | 2.9 | 58.9 | 60.4 | 71.8 |
| *June* | *period 1* | 67.1 | 2.9 | 46.4 | 61.4 | 72.9 |
| *July* | *period 1* | 68.8 | 3.2 | 49.7 | 62.5 | 75.1 |
| *August* | *period 1* | 81 | 4 | 58.2 | 73 | 89.1 |
| *Sep.* | *period 1* | 105.1 | 4.2 | 62.6 | 96.7 | 113.5 |
| *May* | *period 2* | 61.7 | 2.9 | 45.2 | 55.9 | 67.5 |
| *June* | *period 2* | 62.2 | 5.7 | 65.2 | 50.8 | 73.5 |
| *July* | *period 2* | 66.4 | 3.6 | 39.9 | 59.3 | 73.6 |
| *August* | *period 2* | 77.1 | 3.6 | 58 | 69.9 | 84.2 |
| *Sep.* | *period 2* | 91.8 | 4 | 57.4 | 83.7 | 100 |

Table S16: Post-hoc test for the interaction month x period of the linear mixed effect model explaining the variance in BM of male garden dormice.

|  |  | estimate | SE | df | lower CI | upper CI | t | p |  |
| --- | --- | --- | --- | --- | --- | --- | --- | --- | --- |
| period 1 | *June-May* | 1.0 | 2.2 | 69 | -3.4 | 5.4 | 0.5 | 0.64 | n.s. |
| period 1 | *July-May* | 2.7 | 2.5 | 68.9 | -2.3 | 7.7 | 1.1 | 0.29 | n.s. |
| period 1 | *July-June* | 1.7 | 2.6 | 67.5 | -3.5 | 6.8 | 0.6 | 0.52 | n.s. |
| **period 1** | ***August-May*** | **14.9** | **3.2** | **68.8** | **8.6** | **21.3** | **4.7** | **< 0.001** | ******* |
| **period 1** | ***August-June*** | **13.9** | **3.3** | **68.8** | **7.3** | **20.5** | **4.2** | **< 0.001** | ******* |
| **period 1** | ***August-July*** | **12.2** | **3.3** | **60.7** | **5.6** | **18.9** | **3.7** | **< 0.001** | ******* |
| **period 1** | ***Sep.-May*** | **39.0** | **3.6** | **68.9** | **31.9** | **46.0** | **11.9** | **< 0.001** | ******* |
| **period 1** | ***Sep.-June*** | **37.9** | **3.7** | **69** | **30.6** | **45.3** | **10.3** | **< 0.001** | ******* |
| **period 1** | ***Sep.-July*** | **36.3** | **3.8** | **66.1** | **28.7** | **43.8** | **9.6** | **< 0.001** | ******* |
| **period 1** | ***Sep.-August*** | **24.0** | **4.2** | **62.1** | **15.7** | **32.4** | **5.8** | **< 0.001** | ******* |
| period 2 | *June-May* | 0.5 | 5.4 | 67.4 | -10.3 | 11.3 | 0.1 | 0.93 | n.s. |
| period 2 | *July-May* | 4.8 | 3.6 | 48.8 | -2.4 | 11.9 | 1.3 | 0.19 | n.s. |
| period 2 | *July-June* | 4.3 | 6.0 | 63.4 | -7.8 | 16.3 | 0.7 | 0.48 | n.s. |
| **period 2** | ***August-May*** | **15.4** | **3.8** | **58.5** | **7.8** | **23.0** | **4.1** | **< 0.001** | ******* |
| **period 2** | ***August-June*** | **14.9** | **5.3** | **62.2** | **4.4** | **25.4** | **2.8** | **< 0.01** | ****** |
| **period 2** | ***August-July*** | **10.6** | **4.4** | **48.9** | **1.8** | **19.5** | **2.4** | **< 0.05** | ***** |
| **period 2** | ***Sep.-May*** | **30.1** | **4.4** | **58.3** | **21.3** | **38.9** | **6.8** | **< 0.001** | ******* |
| **period 2** | ***Sep.-June*** | **29.6** | **5.9** | **64** | **17.8** | **41.4** | **5.0** | **< 0.001** | ******* |
| **period 2** | ***Sep.-July*** | **25.4** | **5.2** | **54.6** | **15.0** | **35.7** | **4.9** | **< 0.001** | ******* |
| **period 2** | ***Sep.-August*** | **14.7** | **4.3** | **67.4** | **6.2** | **23.3** | **3.4** | **< 0.01** | ****** |
| month_May_ | *period 2-period 1* | -4.4 | 3.1 | 56.8 | -10.6 | 1.8 | -1.4 | 0.16 | n.s. |
| month_June_ | *period 2-period 1* | -5.0 | 6.4 | 64.8 | -17.7 | 7.8 | -0.8 | 0.44 | n.s. |
| month_July_ | *period 2-period 1* | -2.4 | 3.9 | 40.7 | -10.2 | 5.5 | -0.6 | 0.55 | n.s. |
| month_August_ | *period 2-period 1* | -3.9 | 5.2 | 54.6 | -14.3 | 6.5 | -0.8 | 0.45 | n.s. |
| **month_Sep,_** | ***period 2-period 1*** | **-13.2** | **6.2** | **61.6** | **-25.6** | **-0.9** | **-2.2** | **< 0.05** | ***** |

Table S17: Results of the linear mixed effect model, explaining the variance in BM of male garden dormice (n = 83, N = 32) reference level of factors: month_May_, time period_2003-2005_, site_ME,_ random factor = ID. Variance of random effect: 1.47, residual variance: 36.7, R^2^m = 0.77, R^2^c = 0.78. Significant differences are bold.

|  | estimate | SE | df | t | p |  |  |
| --- | --- | --- | --- | --- | --- | --- | --- |
| (Intercept) | 20.9 | 26.1 | 68.1 | 0.8 | 0.43 | n.s. | |
| month_June_ | 1.0 | 2.2 | 69.0 | 0.5 | 0.63 | n.s. | |
| month_July_ | 2.7 | 2.5 | 68.9 | 1.1 | 0.28 | n.s. | |
| **month_August_** | **14.9** | **3.1** | **68.9** | **4.9** | **< 0.001** | ******* | |
| **month_Sep._** | **39.0** | **3.4** | **68.9** | **11.4** | **< 0.001** | ******* | |
| TL | 1.2 | 0.7 | 68.0 | 1.8 | 0.08 | (.) | |
| period 2 | -4.4 | 3.7 | 62.3 | -1.4 | 0.16 | n.s. | |
| site_RL_ | 0.2 | 4.7 | 50.0 | 0.05 | 0.96 | n.s. | |
| site_WK_ | 4.6 | 6.5 | 51.7 | 0.7 | 0.48 | n.s. | |
| T_aminhib_ | 2.7 | 4.3 | 68.9 | 0.6 | 0.53 | n.s. | |
| month_June_: period 2 | -0.5 | 5.8 | 68.1 | -0.1 | 0.92 | n.s. | |
| month_July_: period 2 | 2.1 | 4.2 | 64.6 | 0.5 | 0.62 | n.s. | |
| month_August_: period 2 | 0.5 | 4.6 | 65.6 | 0.1 | 0.91 | n.s. | |
| month_Sep._: period 2 | -8.8 | 5.6 | 66.8 | -1.6 | 0.12 | n.s. | |

Table S18: Tukey post-hoc test for the factor study site of the linear mixed effect model explaining the variance in BM of male garden dormice.

|  | estimate | SE | z | p |  |  |
| --- | --- | --- | --- | --- | --- | --- |
| site_RL_ – site_ME_ | 0.2 | 4.7 | 0.05 | 1.0 | n.s. | |
| site_WK_ – site_ME_ | 4.6 | 6.5 | 0.7 | 0.75 | n.s. | |
| site_WK_ – site_RL_ | 4.4 | 4.5 | 1.0 | 0.58 | n.s. | |

Table S19: Tukey post-hoc test for the linear mixed effect model explaining the variance in TL of adult garden dormice.

|  | estimate | SE | z | p |  |
| --- | --- | --- | --- | --- | --- |
| month_June_-month_May_ | -0.09 | 0.26 | -0.33 | 1.0 | n.s. |
| **month_July_-month_May_** | **0.77** | **0.25** | **3.12** | **< 0.05** | ***** |
| **month_August_-month_May_** | **1.25** | **0.26** | **4.76** | **< 0.001** | ******* |
| month_Sep_-month_May_ | 0.62 | 0.36 | 1.72 | 0.41 | n.s. |
| **month_July_-month_June_** | **0.86** | **0.26** | **3.3** | **< 0.01** | ****** |
| **month_August_-month_June_** | **1.34** | **0.27** | **5.0** | **< 0.001** | ******* |
| month_Sep_-month_June_ | 0.71 | 0.36 | 2.0 | 0.27 | n.s. |
| month_August_-month_July_ | 0.48 | 0.23 | 2.09 | 0.21 | n.s. |
| month_Sep_-month_July_ | -0.15 | 0.32 | -0.49 | 0.99 | n.s. |
| month_Sep_-month_August_ | -0.63 | 0.26 | -2.45 | < 0.1 | (.) |

**
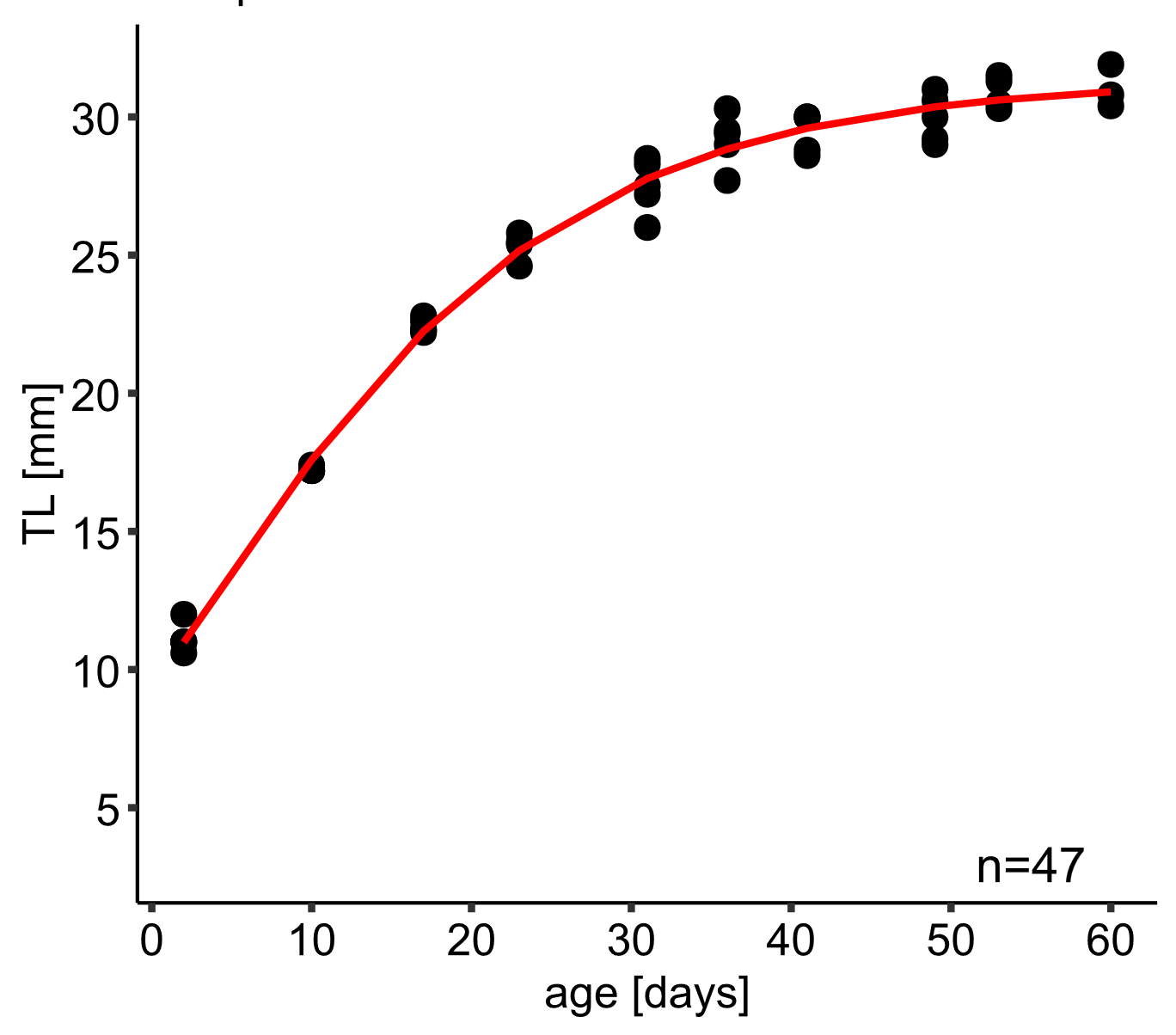
**

Figure S1: Fit of the Gompertz model (line) to the growth data for TL in the reference litter of juvenile garden dormice at the age between 2 and 60 days.
